# Does paternal care influence mate preference? Male and female mating behavior in Threespine Stickleback ecotypes that differ markedly in parental care

**DOI:** 10.1002/ece3.9953

**Published:** 2023-03-28

**Authors:** Rachel H. Corney, Laura K. Weir

**Affiliations:** ^1^ Department of Biology Saint Mary's University Halifax Nova Scotia Canada

**Keywords:** assortative mating, body size, female response, male courtship, sexual selection

## Abstract

Reproductive isolation can occur due to divergence in sexual selection for particular traits. For example, differences in mate preference associated with body size can play an important role in divergence between groups. The importance of mate preference for population divergence may be influenced by other aspects of a mating system, such as the requirement for parental care. In Nova Scotia, Canada, two ecotypes of marine Threespine Stickleback occur sympatrically: a “common” ecotype wherein males provide parental care, and a “white” ecotype that does not exhibit paternal care. The goal of our study was to examine differences in male mate preference between white and common stickleback males to test the prediction that males who invest more in parental care may be more selective about their mates. Because of the link between size and fecundity in this species, we predict that males that invest in parental care should prefer large females, while males that do not provide care will not exhibit preference for larger female size. We found that common male stickleback preferred larger‐bodied females of both ecotypes, while white males showed a preference for larger‐bodied common females. Secondarily, we assessed whether females differed in their willingness to mate with males of different sizes and ecotypes. Common female stickleback had a higher response rate toward smaller white males, which may be associated with their relatively high courtship rates. Counter to previous studies on these ecotypes that suggest that mating is completely assortative, interecotype matings occurred in half of the observed spawning events. This observation, coupled with the results that males may prefer females based mainly on size and females respond to males who court more rigorously regardless of their ecotype, may lend insight into recent genetic evidence for hybridization in the wild.

## Graphical abstract

For this study we tested the prediction that males who provide parental care may exhibit stronger mate preference than those that do not in two ecotypes of the Threespine Stickleback (*Gasterosteus aculeatus*). Our results showed that both male ecotypes tended to prefer the largest females, although males who provide parental care were more consistent with respect to a preference for larger female body size. By contrast, female stickleback generally did not exhibit preference for male size or ecotype, but some of our results suggest that females may prefer smaller males who also court more frequently.

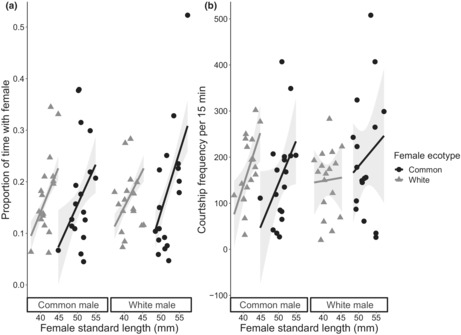

## 1 INTRODUCTION

Sexual selection acts on traits associated with mating success (Hosken & House, 2011). Body size is often a target of sexual selection through both intra‐ and intersexual mechanisms (Andersson & Iwasa, 1996). Directional selection for relatively large body size can occur when males or females fight for access to territories (Kodric‐Brown, 1977; Serrano‐Meneses et al., 2007; van den Berghe & Gross, 1989) and/or mates (Alcock, 1996; Hagelin, 2002; Langston et al., 1990; McCann, 1981). While larger size often leads to success in direct contests because of an association with aggression or fighting ability, it may also be related to an energetic capacity to outlast opponents (Serrano‐Meneses et al., 2007) or court more frequently (Wacker et al., 2012). Furthermore, large size may provide a signal of fertility to potential mates, as it can be related to larger testis size or more sperm in males (Aspbury & Gabor, 2004; Benelli et al., 2016; Gage, 1994; Hagelin, 2002; Hagelin & Ligon, 2001; Poole & Murphy, 2007) and higher fecundity in females (Berglund et al., 1986; Nali et al., 2014; Villa et al., 2018).

In addition to increased reproductive output and advantages during intersexual contests, the strength of sexual selection for larger body size can be influenced by various components of a mating system. In some species, the advantage of large size in intrasexual contests is positively correlated with choice for larger mates because of direct benefits to females and/or indirect benefits via offspring fitness (Andersson, 1994; Côte & Hunte, 1989; Reynolds & Gross, 1992). Furthermore, the degree to which larger individuals gain a reproductive advantage can be associated with fundamental features of a population or species, such as the requirement for parental care of offspring (Emlen & Oring, 1977). In this context, large size may be favored by potential mates because relatively large organisms may be better able to maintain a relatively high body condition for the duration of care to defend eggs and offspring from predators, including conspecifics (Bisazza & Marconato, 1988; Gagliardi‐Seeley & Itzkowitz, 2006).

In addition to providing a signal of mate or parental quality, the size of an individual may affect reproductive decisions. For example, larger individuals of relatively high quality may be more selective about their mates (Amundsen & Forsgren, 2003). Assortative mating based on body size or preference for similar sized mates has been observed in a range of organisms including Water Striders (Arnqvist et al., 1996), American Rubyspots (Serrano‐Meneses et al., 2007), and Pupfish (Kodric‐Brown, 1977). Selection for similarly sized mates may stem from mating constraints such as mechanical barriers that arise because of size differences (Arnqvist et al., 1996), or through sexual selection for mates of a particular size (Nagel & Schluter, 1998). If strong disruptive selection and assortative mating occur (large mating with large and small mating with small), intermediate‐sized individuals may have lower fitness because they have to compete with all individuals in a population, whereas those on the extremes only compete with others in their size class (Kirkpatrick & Ravigné, 2002 and references therein).

Herein, we examine the influence of body size and parental care on mate preference. On Canada's East Coast, two distinct ecotypes of marine Threespine Stickleback occur: a relatively large “common” ecotype that has male parental care, and a smaller “white” ecotype that does not have male parental care (Blouw & Hagen, 1990). The two ecotypes typically occur sympatrically (Blouw & Hagen, 1990), although allopatric populations comprised of only white or common sticklebacks do exist (personal observations). Despite the overlap in breeding habitat, white and common ecotypes are genetically distinct, suggesting that there are characteristics of their breeding biology that may favor assortative mating, although there is still evidence for some gene flow (Samuk, 2016). Behavioral studies have noted that in the field and laboratory the two ecotypes mate completely assortatively (Blouw & Hagen, 1990; Haglund et al., 1990).

In both ecotypes, females require 3 or more days to produce eggs depending on food availability (Wootton et al., 1995 and references therein). These “time out” periods for females can skew the sex ratio toward males during some points in the breeding season. However, male reproductive availability can also affect the intensity of sexual selection, because common male Threespine Sticklebacks provide parental care (Blouw, 1996; Jamieson et al., 1992a; van Iersel, 1953). During the paternal care period, common males are not available to mate for 7 or more days (Wootton et al., 1995). Thus, these time‐outs can shift the sex ratio of available mates. The relatively equal reproductive availability of male and female Threespine Sticklebacks suggests a mutual mate choice system for many ecotype pairs and populations (Blouw & Hagen, 1990; Kraak & Bakker, 1998; Rowland, 1982), although female choice is designated as the most prominent mate choice system (Bakker, 1993; Baube et al., 1995; Bay et al., 2017; Conte & Schluter, 2013; McPhail, 1969; Milinski & Bakker, 1990). In contrast to common males, white male Threespine Sticklebacks do not provide parental care, but rather disperse eggs from their nest after fertilization (Blouw, 1996; Jamieson et al., 1992b) and thus avoid or markedly shorten any reproductive “time‐outs.” Therefore, we expect that sex ratio in the white Threespine Sticklebacks may be male‐biased throughout the mating season and that male mate choice may not occur in this ecotype, or it may be relatively less important to the mating system than in common Threespine Stickleback.

The main goal of our study was to examine differences in mate preference between white and common stickleback males to test the prediction that males who invest more in parental care may be more likely to be selective about their mates. Secondarily, we also assessed whether females exhibited differences in willingness to mate with males of different sizes and ecotypes.

## 2 METHODS

### 2.1 Sampling sites and fish collection

Fish collection occurred in June 2020 at three sites located across Nova Scotia, Canada: Rainbow Haven Estuary (44.654482°N, −63.421073°W), Antigonish Landing (45.632433°N, −61.960333°W), and Crossing Road (45.442708°N, −61.50575°W). All three locations are brackish water environments supplied by the Atlantic Ocean, with a salinity range of ~18 to ~30 ppt and temperature ranging between ~13 and ~21°C. However, the three locations differ environmentally; Antigonish Landing (AL) is characterized by muddy substrate and is relatively bare of large rocks and algae; Crossing Road (CR) has a rocky substrate surrounded by patches of filamentous algae such as *Cladophora* spp.; Rainbow Haven Estuary (RHE) is intermediate between the other two sites with a combination of a muddy and rocky substrate surrounded by filamentous algae and tall grass. The presence or absence of these environmental factors is important as Threespine Sticklebacks have specific habitat requirements for nesting sites, wherein common males build nests in muddy substrate, and white males use filamentous algae as their main nesting material (Blouw & Hagen, 1990).

A total of 120 fish (AL: 15 male and female common Threespine Stickleback; CR: 18 male and female white Threespine Stickleback; RHE: 12 male and female common and 15 male and female white Threespine Stickleback) were collected using a combination of unbaited Gee's minnow traps (1/4 inch mesh) for female stickleback and easily identifiable males (nuptial colouration visible) and dip‐netting males on their nests by hand. Fish were identified visually in the field for sex and ecotype by observing their size, colouration, abdominal shape, and nesting location, as these traits vary between the sexes and ecotypes (Blouw & Hagen, 1990). After identification, fish were transported to the Saint Mary's University Aquatic Facility, where they were held in 15‐gallon stock tanks with the sexes and ecotypes housed separately. Stock tanks were maintained at a water temperature of 20–22°C, salinity of 15 ppt ± 1 ppt, and a light cycle of 16‐h light:8‐h dark to reflect breeding conditions in the wild (Blouw & Hagen, 1990). All stock tanks contained gravel, artificial plants, structures for refuge, algae collected from Rainbow Haven estuary for nesting material, and a waterfall filter for aeration. Fish were fed a diet of frozen *Mysis* shrimp and bloodworms once a day.

### 2.2 Experimental design

**FIGURE 1:**
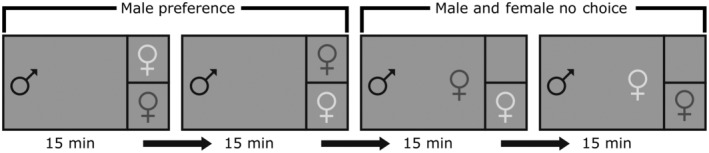
Experimental setup for the male preference and male and female no‐choice experiments. The larger part of the tank contained the focal male fish and nest‐building materials. The two smaller portions of the tank contained one female each for the first part of the male preference trial. For the second part of the experiment, the male and female no‐choice trials, one of the two females was released into the larger portion of the tank with the male. Each event consisted of a 15‐min interval totaling a 1‐h‐long experimental trial. The black horizontal line indicates an opaque partition separating the female fish visually from one another, the black vertical line in indicates a clear partition separating the male and female fish physically from one another.

The experimental setup consisted of six 10‐gallon focal tanks that were held under the same salinity, temperature, and photoperiod as the stock tanks. Tanks were partitioned into three compartments: one large compartment comprising three‐quarters of the tank that held the focal male stickleback (either common or white) and nesting material (both algae and sand); the other two compartments were evenly divided into the remaining one‐quarter of tank space and housed one common and one white female separately (Figure 1). This arrangement was used to minimize any effects of female proximity to a nest that may occur if females were on opposite ends of the tank in a more traditional setup and to reduce chemical communication among the fish, though there was still the potential of minimal waterflow among the compartments at the connection points of the plexi‐glass barrier.

A trial consisted of two stages of choice experiments that were conducted sequentially: a partitioned stage and an interaction stage. Prior to the start of a trial, males were transferred to an experimental tank by hand‐net that contained nesting material and were allowed from 1 to 4 days to construct a nest; males that did not build nests were replaced. Nest material preferences of common and white stickleback have been previously reported: common stickleback prefer sand and white stickleback prefer algae (Blouw & Hagen, 1990; Jamieson et al., 1992a), but both ecotypes may build nests using both materials (Blouw & Hagen, 1990; Corney, 2019). Nest building behaviors were recorded during this study, though not the focus. Both male stickleback ecotypes readily build nests in a laboratory setting (personal observation). Nests were identified as an opening in a constructed mound made of algae and/or sand held together by a glue substance produced by their kidneys (Blouw & Hagen, 1990; Jakobsson et al., 1999; Jamieson et al., 1992a). These observations acted as a confirmation of the visual identification of male ecotype in the field. Following successful nest construction, one female of each ecotype was transferred by hand‐net into each of the smaller compartments. Females and the focal male were relatively size‐matched as closely as possible using visual estimation to avoid any stress associated with handling to collect body size measurements. Large common females were paired with large white females and small common females with small white females; and within trials the common female was always larger than the white female. Both males and females varied by ecotype (white and/or common) and in geographical distribution (population type: sympatric and/or allopatric population).

Each stage lasted 30 min for a total of 1 h. During the partitioned stage, all three fish remained in their respective compartments; female position was switched after 15 min to account for any inherent side preference for the male. During the interaction stage, a female selected at random was released from her compartment to interact with the male for 15 min, at which time that female was returned to her compartment and the other female released in the compartment with the male for the same duration. Both females were visible to the focal male for the entire trial.

All trials were recorded with an Enviro R jvc camcorder (GZ‐R460D model). After a trial was complete, the male and the two females were tagged using subcutaneous visual implant elastomer tags (Northwest Marine Technologies) for individual fish identification before returning them to their respective stock tanks. Body size was recorded as standard length to the nearest 0.01 mm and was collected for two purposes: first, to examine the influence of body size on Threespine Stickleback mate preference; and second to confirm the ecotype of the fish based on size distributions of fish in the experiments. Data for the body size measurement of the fish trios can be found in Table A3 of the Appendix.

There were a total of 35 observational trials: 18 replicates with a common male and 17 replicates with a white male. Male fish were only used once per mate choice trial, while female fish were used one to four times (mean = 1.79 ± 0.15 SE). Individual female identity was included in our statistical analyses to account for the fact that they were used multiple times. Female fish were reused because gravid females were required to invoke a male response. Gravidity of females was assessed by their distended abdomen. A total of 39 female fish and 35 male fish were used during the experiment.

### 2.3 Behavioral observations

To investigate mate preference of male and female Threespine Sticklebacks, video recordings were uploaded to JWatcher (version 1.0), a quantitative behavioral analysis program (Blumstein et al., 2020). JWatcher allows for the observer to record events to assess the frequency and time allocated to specific behaviors conducted by the focal subject.

#### 2.3.1 Male preference

During the partitioned stage, male preference was quantified by measuring the amount of time a male spent in proximity to each of the contained females. Proximity was determined when the male was located directly in front of the clear plexiglass barrier of a female's compartment. If the male was located elsewhere within the tank, no recording of time spent in proximity to a female was conducted. Recording was initiated each time the male “poked” the clear plexiglass barrier with his mouth (“glass poking”) and ceased when he stopped the behavior for more than 1 s or retreated from the barrier. This method was selected over the male being in the vicinity of the female to ensure the male was actively attempting to engage with the female and not simply resting in front of the barrier.

During the interaction stage, male preference was assessed as courtship frequency, which was determined by counting different courtship behaviors conducted toward the released female during the interaction stage. Six courtship behaviors were observed and recorded. Four of the six behaviors were shared between both white and common males (zig‐zagging, biting, leading, and nest poking), while dorsal pricking was only observed for common males and side fanning for white males. Zig‐zagging is a stereotypical courtship behavior observed in Threespine Stickleback and occurs when the male quickly swims in one direction then shifts direction to form a Z‐pattern. Biting was recorded as a unique event each time a male touched the female on her ventral or lateral surface with an open mouth. When a male was observed swimming away from a female directly to his nest, it was recorded as one instance of leading. Nest poking was characterized by a male poking his nest with his snout. Each poking action was recorded as an individual behavior even if many occurred in succession. During dorsal pricking, the male uses his dorsal spines to poke the female's abdomen; as noted above, this occurs only in common males. For white males only, side fanning was identified as each time the male turned horizontally and moved in an undulated fashion. Side fanning was recorded only once when the male turned on horizontally, regardless of the duration of the behavior. After a trial, the frequency for each of the behaviors were summed to calculate total courtship frequency for analysis.

#### 2.3.2 Female response

Only the interaction stage was used to assess female response. This was a binomial variable associated with whether or not she conducted any of the typical “head‐up,” “follow,” and/or “inspect nest” response behaviors that characterize female willingness to mate when released with the male. A head‐up display was identified when the female pointed her head slightly upward and displayed her abdomen to the male. Following was recorded when the female accompanied the male to his nest. Lastly, nest inspection occurred when the female placed her snout in the nest opening but did not enter the nest.

#### 2.3.3 Spawning

The pairs were also observed for spawning events. Spawning in stickleback is identifiable when the female enters the nest (to deposit her eggs) followed by the male entering the nest to fertilize eggs as the female departs. This was recorded as a spawning event only if the female's abdomen had decreased markedly in size when she emerged from the nest. When spawning occurred, the time that the female and male entered and left the nest were recorded. If spawning took place, behavioral recording for that portion of the interaction stage was terminated and nests were visually checked for the presence of eggs to confirm spawning. If spawning occurred during the first trial (three of 10 events, one of which occurred after the 15‐min period), the second female was still exposed to the male during the interaction stage. Assortative spawning was determined based on whether the male and female were of the same (pure cross) or different (hybrid cross) ecotype.

### 2.4 Statistical analyses

All analyses were implemented using the statistical software R v4.0.2 (R Core Team, 2020) and analyzed using a model comparison method utilizing Akaike Information Criteria (AIC). For all analyses, all possible models were compared and ranked using Akaike Information Criteria corrected for small sample size (AICc) using the dredge function in the “MuMIn” package, version 1.43.17 (Barton, 2020). Models were assessed in two ways: (1) by examining the residuals versus the fitted values using the “plot” function; and (2) by calculating the dispersion parameter if models were fit using the Poisson or negative binomial distributions. To quantify the fit of the best models to our data, we calculated the pseudo‐*R* ^2^ values using the r.squaredGLMM function in the MuMin package (Barton, 2020). All pseudo‐*R* ^2^ values reported in the Results are marginal values, and thus are based only on the fixed predictors, excluding the random effects.

#### 2.4.1 Male preference: ecotype and female body size

Male preference was examined in each phase of the trials, first by assessing the proportion of time a male spent with each female during the partitioned stage and second by examining the number of courtship behaviors he conducted toward each female during the interaction stage. We used generalized linear mixed models (GLMM) to analyze the fixed effect of male ecotype, female ecotype, female body size, and their interactions on measurements of male preference. Individual males were included as a random effect to account for each male being able to associate with both a white and a common female during this stage. Similarly, because 20 of 39 females were used in more than one trial, individual female identity was also included as a random effect. Female body size was standardized for analyses because of the large variation in standard length using the scale function in base R. The scale function is a standard Z‐transformation that is calculated for each data point by subtracting the mean and dividing by the standard deviation.

Preference during the partitioned stage was measured as the time that the male was in proximity to each female divided by the total observation time (30 min). We modeled errors for these data using Gaussian error distribution, as they do not represent true proportions, and comparisons of model fits using residuals for both approaches indicated that the residuals were normally distributed. Data for the frequency of male courtship during the interaction stage were corrected for time to account for the fact that nine of the 35 trials did not last the full 15‐min observation period because spawning occurred earlier in the time period (two during the first interaction stage and seven during the second interaction stage). To do this, we divided the typical observation time (15 min) by the duration of the trial and then multiplied that value by the behavioral frequency to obtain an equivalent for a 15‐min time period. The final value was rounded to the nearest integer to approximate count data. To analyze these data, we modeled errors using a Poisson distribution because these data were recorded as counts. We examined the fit of these models by examining the dispersion parameter; if this was reasonably close to a value of 1.0, we considered this a good fit for the Poisson (or negative binomial) distribution. Furthermore, a two‐sample *t*‐test was conducted to determine whether there was an order effect on males courtship rate toward the first released female compared with the second. Results indicated that there was no order effect on male courtship rate and males did not court the first released female more vigourously than the second female (*p* = .78, *t* = −0.28, df = 68).

In addition to female size as a potential indicator of male preference, we examined whether the body size difference between the sexes may be related to the frequency of male courtship as a potential measure of size matching (size matching has been shown in limnetic and benthic sticklebacks) (Conte & Schluter, 2013; Nagel & Schluter, 1998). For this analysis, we calculated size difference by subtracting the male size from the female size for both females the male interacted with during a trial. This difference in size was then used as a continuous fixed effect while the response variable and random effects remained the same as the previous analysis.

#### 2.4.2 Female response: ecotype and male body size

Female response was recorded as a binary variable with a value of 0 (no response) or 1 (response). To be assigned a value of 1, the female followed the male, inspected his nest, or performed a stereotypical “head up” display. A GLMM with a binomial error distribution with female response as the binary response variable was used to determine the effects of male ecotype, female ecotype, scaled male body size, and their interactions on female response. As before, individual males and individual females were included as random effects and male body size was scaled to account for the large variation in stickleback standard length associated with ecotype. Similar to the analysis for male preference, we examined whether there were any indications of size matching, and thus we included an analysis with size difference as a fixed effect, while the response and random effects remained the same.

## 3 RESULTS

### 3.1 Male preference

**FIGURE 2:**
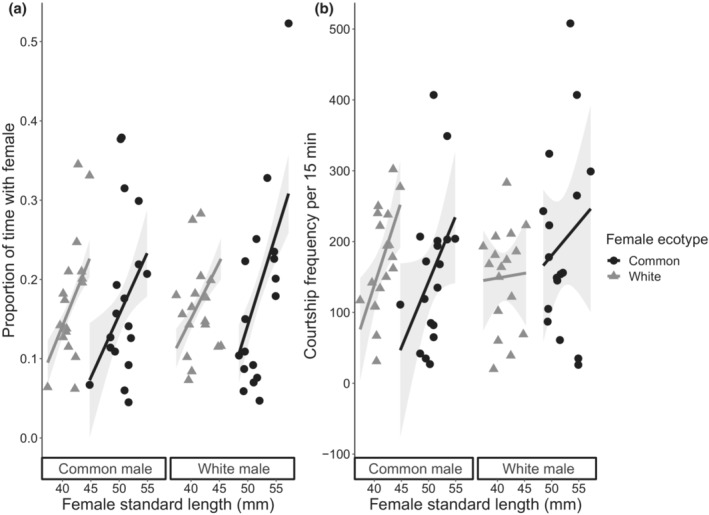
(a) Proportion of time spent in proximity to and (b) total courtship frequency of common (left) and white (right) male Threespine Sticklebacks toward common (black) and white (gray) female sticklebacks based on female standard length (mm) during the 15‐min interaction stage. Black circles and gray triangles represent the frequencies for individual males. Individual males are counted twice as each male had an observation recorded for both a common and white female stickleback (*n* = 35). Regression lines are best fit based on the top model, and gray shaded areas are the standard error of the estimates.

Both the proportion of time a male spent in proximity to a female and the frequency of courtship behavior directed toward a female were associated with female length (Table 1; Figure 2). There were five models that fell within our criteria as a reasonable fit to the data for the proportion of time spent near a female. While the outcome of this analysis was not definitive, all five models shared the common factors of female size and female ecotype (Table 1). In all cases, the coefficient related to the continuous dependent variable of body size was positive, suggesting that both common and white male Threespine Stickleback increased the amount of time spent in proximity to larger females (Table 1; Figure 2a). The two models for courtship frequency retained interactions between the predictors, particularly female size and male ecotype; this indicates that white and common males exhibit different relationships between courtship frequency and female size. The first model had two interaction terms: male ecotype × female ecotype and male ecotype × female size. The second model retained the same two interaction terms as the first model with an additional interactive effect between female ecotype and female size (Table 1). Dispersion parameters for both models were close to 1 (1.05 and 1.06, respectively). Interestingly, white male stickleback courtship rate increased with female size for common females to a larger extent than they did for white females, which is a driver of the interactions in our top models. In comparison, common male stickleback courted larger females at a higher frequency than smaller females, but did not show a continuous preference with body size (i.e., they courted large white females at a higher frequency than small common females, despite the fact that small common females are larger than the largest white females; Figure 2b).

**TABLE 1:** Generalized linear model selection for the effects of scaled female size, male ecotype (common or white), and female ecotype (common or white) on the proportion of time spent in proximity to a female during the first phase of the experiment and the adjusted total courtship behavior frequency conducted toward the released female by male Threespine Sticklebacks.

Response variable	Predictor	*k*	AICc	∆AIC	*ω*_*i*_	Pseudo *R* ^2^
Proportion of time	**Female ecotype + female size**	**6**	**−141.4**	**0.00**	**0.203**	**.20**
	**Female ecotype × male ecotype × female size**	**11**	**−140.8**	**0.60**	**0.150**	**.32**
	**Female ecotype + male ecotype + female size**	**7**	**−140.8**	**0.63**	**0.148**	**.22**
	**Female ecotype × female size**	**7**	**−140.5**	**0.90**	**0.130**	**.23**
	**Female ecotype × female size + male ecotype**	**8**	**−140.5**	**0.99**	**0.124**	**.25**
Total courtship behavior	**Female ecotype × male ecotype + male ecotype × female size**	**8**	**874.2**	**0.00**	**0.619**	**.12**
	**Female ecotype × male ecotype + female ecotype × female size + male ecotype × female size**	**9**	**875.7**	**1.43**	**0.302**	**.12**
	Female ecotype × male ecotype × female size	10	878.3	4.11	0.079	
	(Intercept)	3	921.7	47.52	0.000	
	Female ecotype × female size	6	922.8	48.61	0.000	

*Note*: Shown above are the number of predictors (*k*), Akaike Information Criterion corrected for small sample sizes (AICc), the difference between the model with the lowest AICc value compared to all other predictors (∆AICc), *ω*_*i*_, the weight of a model relative to the complete model set (*n* = 19), and the marginal pseudo‐*R* ^2^ value for top models. The bolded models are those with the lowest AICc values by a difference of two or more. Only the top five models are shown; to view the complete table see the associated Dryad file (Table A1). Models with interactions also contain lower order main effects for the predictors.

### 3.2 Female preference

**FIGURE 3:**
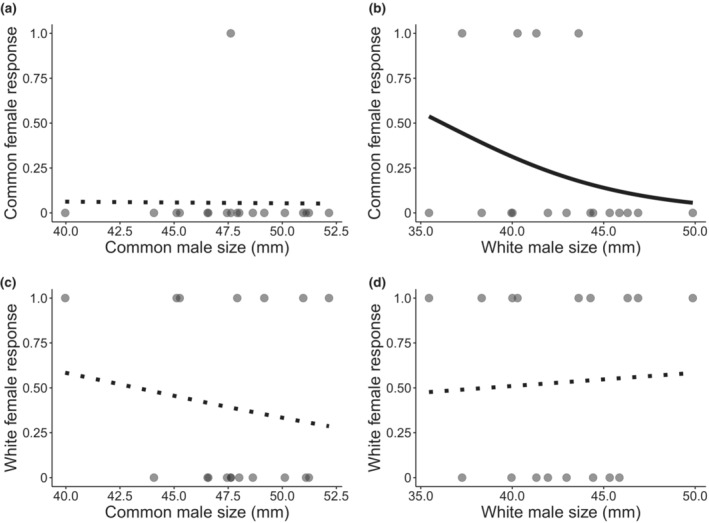
Probability of response of common females toward (a) common (*n* = 18) and (b) white (*n* = 17) male Threespine Stickleback and white females toward (c) common (*n* = 18), and (d) white (*n* = 17) male Threespine Stickleback of different standard length (mm). Gray circles represent individual females. Dashed lines denote relationships that include only the intercept, while solid lines indicate that male size is included in the best models.

Results for female preference were ambiguous as multiple models fit our selection criteria; all three predictors influenced the response of females (Table 2). Because of the interactive effect between female ecotype and male size, we also examined patterns within subsets of our data by constructing four additional models using subsets of the possible female and male ecotype combinations. The influence of male size on the probability of female response was only retained in our top models when common females were in the presence of white males (Table 2; Figure 3b), whereby common female stickleback were more likely to respond to smaller‐bodied white male stickleback (Table 2; Figure 3b). However, common females were overall less likely to respond during courtship compared with white females (Figure 3).

**TABLE 2:** Generalized linear mixed model selection for the effects of scaled male size, male ecotype (common or white), and female ecotype (common or white) on the probability of female response to male Threespine Stickleback.

Ecotype combination	Predictor	*k*	AICc	∆AICc	*ω*_*i*_	Pseudo *R* ^2^
Both female and male ecotypes	**Female ecotype + male ecotype**	**5**	**84.9**	**0.00**	**0.199**	**.18**
	**Female ecotype + male size**	**5**	**85.0**	**0.07**	**0.192**	**.17**
	**Female ecotype**	**4**	**85.3**	**0.45**	**0.159**	**.13**
	**Female ecotype × male size**	**6**	**86.1**	**1.26**	**0.106**	**.23**
	**Female ecotype + male ecotype + male size**	**6**	**86.6**	**1.76**	**0.082**	**.19**
	**Female ecotype × male ecotype**	**6**	**86.9**	**1.98**	**0.074**	**.21**
Common female and common male	**(Intercept)**	**2**	**8.0**	**0.00**	**0.811**	**—**
	Common male size	3	10.9	2.91	0.189	
Common female and white male	**(Intercept)**	**2**	**23.4**	**0.00**	**0.668**	
	**White male size**	**3**	**24.8**	**1.40**	**0.332**	**.11**
White female and common male	**(Intercept)**	**2**	**20.0**	**0.00**	**0.811**	**—**
	Common male size	3	22.9	2.91	0.189	
White female and white male	**(Intercept)**	**2**	**28.4**	**0.00**	**0.813**	**—**
	White male size	3	31.3	2.94	0.187	

*Note*: The first output is the full model, while all others are specific subsets. Shown above are the number of parameters (*k*) for the predictors, Akaike Information Criterion corrected for small sample sizes (AICc), the difference between the model with the lowest AICc value compared to all other predictors (∆AICc), *ω*_*i*_, the weight of a model relative to the complete model set (*n* = 19 and 2, respectively), and the marginal pseudo‐*R* ^2^ value for top models. The bolded models are those with the lowest AICc values by a difference of two or more. Only the top five models are shown, except in the case of the first reported model where the top six are shown for completeness; to view the complete table see the associated Dryad file (Table A2). Models with interactions also contain lower order main effects for the predictors.

### 3.3 Body size effects on male and female behavior

**FIGURE 4:**
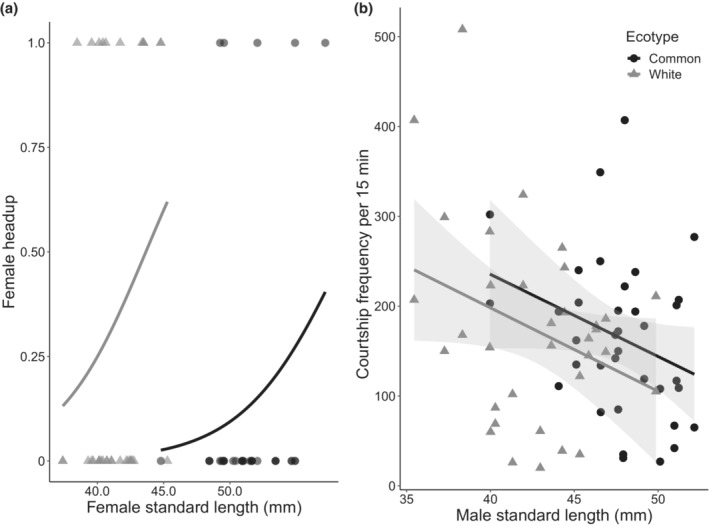
(a) Headup propensity for females based on female standard length (mm; *n* = 35). (b) Total courtship frequency of common (black) and white (grey) male Threespine Sticklebacks towards the released female stickleback based on male standard length (mm) during the 15‐min interaction stage (*n* = 35). Black circles and grey triangles represent the frequencies for individuals. Lines are based on best fit models; grey represents the white ecotype and black represents the common ecotype. Individual males are shown twice as each male had an observation recorded for both a common and white female stickleback.

Our results for male preference and female responses indicated that there was an influence of the body size of potential mates on courtship effort or response propensity. Thus, we conducted post hoc examinations of the relationships between body size and behavior. Because male courtship frequency may be related to female head‐up displays (Bakker & Rowland, 1995; Rowland, 2000), we first examined the relationship between the propensity for females to perform head‐up displays and female body size as a potential factor that could influence our result. This analysis included female ecotype and female body size as fixed effects, and female identity as a random effect. We found that female ecotype had an effect on the propensity to perform head‐up displays (white females: 12 of 35 trials; common females: 5 of 35 trials). In addition, the best model retained an effect of female size, indicating that larger females of each ecotype may have been more likely to perform head‐up displays in comparison to smaller ones (Table 3; Figure 4a).

**TABLE 3:** Generalized linear model selection for the effects of scaled body size and ecotype (common or white) on the propensity to perform head‐up displays (for females) and adjusted total courtship behavior frequency (for males) in the second phase of the experiment.

Response variable	Predictor	*k*	AICc	∆AICc	*ω*_*i*_	Pseudo *R* ^2^
Female response	**Ecotype + female size**	**4**	**78.2**	**0.00**	**0.46**	**.13**
	**Ecotype**	**3**	**79.8**	**1.59**	**0.21**	**.06**
	Ecotype + female size	5	80.5	2.30	0.14	
	(Intercept)	2	80.8	2.57	0.13	
	Female size	3	82.2	4.01	0.06	
Male courtship frequency	**Male size**	**4**	**833.7**	**0.00**	**0.414**	**.06**
	**Male ecotype + male size**	**5**	**834.9**	**1.24**	**0.223**	**.07**
	**(Intercept)**	**3**	**834.9**	**1.28**	**0.219**	**—**
	Male ecotype	4	837.1	3.42	0.075	
	Male ecotype × male size	6	837.2	3.59	0.069	

*Note*: Shown above are the number of parameters (*k*) for the predictors, Akaike Information Criterion corrected for small sample sizes (AICc), the difference between the model with the lowest AICc value compared to all other predictors (∆AICc), *ω*_*i*_, the weight of a model relative to the complete model set (*n* = 5), and the pseudo‐*R* ^2^ value for top models. The bolded models are those with the lowest AICc values by a difference of two or more. Models with interactions also contain lower order main effects for the predictors.

Second, we investigated whether males of different sizes differed in their courtship effort. We used scaled male body size, male ecotype and their interaction as the fixed effects, individual males as the random effect, and total courtship frequency as the response variable. A negative binomial error distribution was used due to overdispersed count data (dispersion parameters: Poisson distribution = 4.26; negative binomial distribution = 1.03). While the effect of body size and ecotype on courtship frequencies were weak (three models, including the one with only the intercept as a predictor, fit our selection criteria), we found that courtship frequency of male Threespine Stickleback is influenced primarily by male body size, or an additive relationship between body size and ecotype. Smaller‐bodied common and white male stickleback tend to court at a higher frequency than larger‐bodied males (Table 3; Figure 4b), with common males exhibiting higher courtship frequency in this experiment.

### 3.4 Size matching and spawning

**FIGURE 5:**
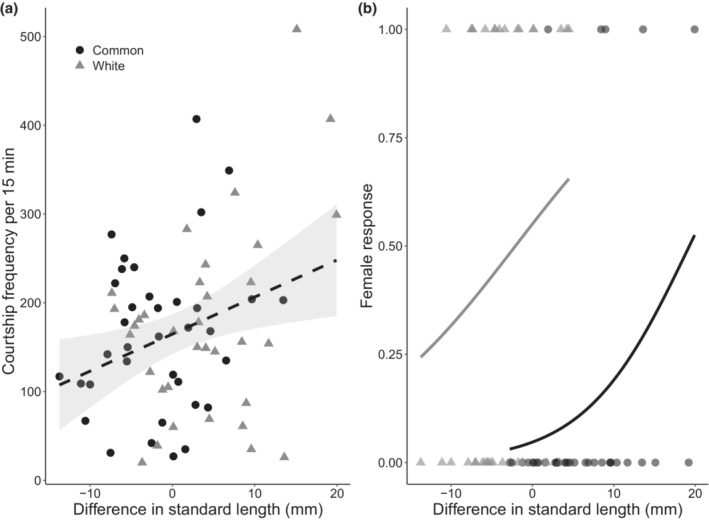
(a) Total courtship frequency towards the released female by both common(black) and white (grey) male Threespine Sticklebacks (*n* = 35) and (b) probability of common (black) and white (grey) female Threespine Stickleback response to male stickleback (*n* = 70) with reference to the difference in standard length (mm) between the courting male and the released female. Values above zero on the *x*‐axis indicates that the female was larger than the male, while for values below zero the male was larger. Black circles and grey triangles represent individual (a) males and (b) females. Individual males are counted twice as each male had an observation recorded for both a common and white female stickleback in a trial. Regression lines are best fit based on the top model, and grey shaded areas are the standard error of the estimates. Dashed lines represent uncertain relationships, while solid lines are relationships that were unequivocally the best in our analysis.

We did not find strong evidence for size matching in our experiment. Our results revealed that male courtship frequency was very weakly associated with the size difference between the courting male and released female (Table 4, Figure 5a). Regardless of ecotype, male Threespine Stickleback courted females that were longer in standard length than themselves at a higher rate than females that were shorter (Figure 5a). In comparison, there was an effect of ecotype and size difference on the likelihood of female response, whereby white females responded more than commons, and females of both ecotypes were more likely to respond to males who were relatively larger than themselves (Table 4; Figure 5b).

**TABLE 4:** Generalized linear model selection for the effects of differences in body size and ecotype (common or white) on the adjusted total courtship behavior frequency (for males) and the propensity to perform reproductive behaviors (for females) in the second phase of the experiment.

Response variable	Predictor	*k*	AICc	∆AICc	*ω*_*i*_	Pseudo *R* ^2^
Male courtship frequency	**(Intercept)**	**3**	**921.7**	**0.00**	**0.48**	**—**
	**Body size difference**	**4**	**923.3**	**1.54**	**0.22**	**.02**
	Ecotype	4	923.9	2.19	0.16	
	Size difference + ecotype	5	925.2	3.51	0.08	
	Size difference × ecotype	6	926.3	4.59	0.05	
Female response behavior	**Size difference + ecotype**	**5**	**83.0**	**0.00**	**0.59**	**.21**
	Size difference × ecotype	6	85.1	2.10	0.21	
	Ecotype	4	85.3	2.37	0.18	
	(Intercept)	3	7.47	7.47	0.01	
	Size difference	4	9.64	9.64	0.01	

*Note*: Shown above are the number of parameters (*k*) for the predictors, Akaike Information Criterion corrected for small sample sizes (AICc), the difference between the model with the lowest AICc value compared to all other predictors (∆AICc), *ω*_*i*_, the weight of a model relative to the complete model set (*n* = 5), and the pseudo‐*R* ^2^ value for top models. The bolded models are those with the lowest AICc values by a difference of two or more. Models with interactions also contain lower order main effects for the predictors.

Ten spawning events occurred during the interaction phase of the 35 mate choice trials (28.6%). Five of the 10 spawnings were hybrid matings; four of these were between common males and white females. Of the five pure crosses, one was a common male and female cross, while four were between a white male and female. The pure crosses differed in size by an average of 3.93 mm, with the common female being larger than the common male. In three of the four pure white stickleback crosses, the male was larger than the female. The hybrid crosses size differences were almost double the pure crosses, averaging 6.61 mm with the common stickleback of both sexes being larger than their white stickleback mating partner.

## 4 DISCUSSION

Our results did not fully support our prediction that males who invest more in parental care may be more likely to be selective about their mates by showing more interest (i.e., higher courtship frequency) toward a female of their own ecotype. If our prediction had been supported by the data, common males should not only prefer larger females, but this relationship should have been linear rather than additive (i.e., no effect of ecotype as common females are larger than white females). While in general our analyses indicated that male stickleback preferred larger females, for both of our measures of male preference (proportion of time with a female and courtship frequency), the best models also included the effects of male and female ecotype. In addition, we found that larger females were more likely to perform head‐up displays, which may influence male courtship independently of body size (Bakker & Rowland, 1995; Rowland, 2000). For female stickleback, common females were more likely to respond to smaller‐bodied white male stickleback, which may be associated with the tendency for small males to court at a higher frequency than large males. White females did not exhibit preference for males of a particular ecotype or size.

### 4.1 Male preference and female body size

The Threespine Stickleback mating system is typically described as female choice‐dominated for the common ecotype and various other stickleback ecotypes and populations including hybrids (Bakker, 1993; Baube et al., 1995; Bay et al., 2017; Conte & Schluter, 2013; McPhail, 1969; Milinski & Bakker, 1990). While male mate choice is often recognized as a component of the stickleback mating system (Bakker & Rowland, 1995; Kraak & Bakker, 1998; Rowland, 1982, 1989b), male selection for identifiable female traits may be difficult to discern. Indicators of female quality focus mainly on body size in relation to fecundity in various populations (Hagen, 1967; Rowland, 1989b; Wootton, 1973). For this study, female stickleback were not randomly selected for a trial, but rather females were matched based on relative size matching. When a large common female was selected for a trial, a large white female was selected as well; however, common female stickleback were always larger in size than the white female within the same trial. The male ecotypes in this study differ in body size, but may overlap at the extremes (small common stickleback compared to a large white stickleback, data from this study, see Table A3 in the appendix). Within the ecotypes, size differences have been observed between male and female common sticklebacks, with females being larger (Blouw & Hagen, 1990). This size dimorphism between the two sexes may have a connection to the fecundity of females. In common, stickleback fecundity varies with female body size (Rowland, 1989b), with larger females producing more or heavier eggs (Hagen, 1967; Wootton, 1973) as seen across stickleback populations. Common male stickleback from North American and Switzerland populations have been shown to prefer larger bodied and more distended females (Kraak & Bakker, 1998; Rowland, 1989b), which allows for males to distinguish between “high‐quality” and “low‐quality” females (Kraak & Bakker, 1998) and common males did increase courtship for larger females in this study as did white males (for common females). While body size was a potential predictor of male courtship, there is an additional component in our study that may be related to female behavior, as larger females were more likely to exhibit head‐up behavior, which is a potential correlate with male courtship (Bakker & Rowland, 1995; Rowland, 2000). A detailed, controlled analysis of this phenomenon is a potential avenue for future study.

Within the white stickleback, similarly sized females produce a variety of clutch sizes (Blouw, 1996), potentially indicating that body size may not be the best correlate of fecundity for this ecotype, which may be reflected in the lack of size preference demonstrated by white males when in the presence of white females in our study. White male preference for larger common females, despite differences in ecotype, may arise from the potential cost of courtship behaviors and/or signals. A study conducted on *Astatotilapia flaviijosephi*, a maternal mouthbrooding cichlid species, found that males were selective toward the female they courted even though they provided no paternal care (Werner & Lotem, 2003). The authors proposed that this was due to the potential cost of courtship through energy expenditure and predation risk, as well as the risk of sperm depletion (Werner & Lotem, 2003). Additionally, in another fish species where female fecundity varies with body size, *Girardinichthys viviparus*, males exhibited choosiness toward females by performing more and longer displays toward larger, more distended, and more orange females (Méndez‐Janovitz & Constantino, 2017). Male *G. viviparus* courtship is considered costly as they must remain with only one female for an extended period of time, thus reducing their potential for copulations with other females (Méndez‐Janovitz & Constantino, 2017). The gravidity of females and male energy expenditure and sperm reserves were not measured in this study, and future avenues of work may involve quantifying the relationship between female fecundity, male courtship costs, and sperm depletion in the white stickleback.

Mating based on body size is not uncommon in the Threespine Stickleback species complex. Limnetic and benthic Threespine Stickleback ecotypes are known to interbreed, and in some instances, they differentiate between one another by size matching (Nagel & Schluter, 1998); however, when the two ecotypes are within similar sizes to each other hybridization may occur (Conte & Schluter, 2013; Nagel & Schluter, 1998). Additionally, populations of anadromous and freshwater Japan Sea and Pacific Ocean ecotypes of Threespine Stickleback also show some variation in courtship behaviors toward differently sized individuals (Ishikawa & Mori, 2000). Moreover, some male fish differentially allocate their sperm production, by producing and/or allocating more sperm in the presence of larger‐bodied females than smaller females (Aspbury & Gabor, 2004; Marconato & Shapiro, 1996; Shapiro et al., 1994). However, Threespine Stickleback males from the Netherlands do not adjust their ejaculate size nor their sperm investment for females of differing length, weight, or egg mass, but that larger males invested more sperm than smaller males (Zbinden et al., 2001).

Previous studies have reported that male white Threespine Sticklebacks court at a higher frequency than common male stickleback (Haley et al., 2019; Jamieson et al., 1992a). In this study, we found that common males generally courted females at a higher rate than white males (shown in Figure 4). The difference in courtship rate between common and white male stickleback has been attributed to the fact that common males perform paternal care and must allocate more energy toward parental care behaviors (van Iersel, 1953), while noncaring white males can allocate their energy toward courtship or other behaviors or traits (e.g., colouration). During this experiment, common males had no eggs present in their nest for the duration of the trials and did not need to perform parental care behaviors, allowing males to divert their efforts toward courting females. This increase in courtship could be a signal of male quality as has been suggested in other studies that have reported that courtship intensity could be a stronger signal of quality than the presence of eggs in a male's nest that act as a primer for males to court more (Jamieson & Colgan, 1989). Similarly, males who court more have relatively higher offspring survival rate after hatching as seen in a population from Spain (Chiara et al., 2022).

### 4.2 Female response and male body size

Previous studies of common females from various populations have documented preference for larger common males (Kraak & Bakker, 1998; Moodie, 1982; Rowland, 1989a). This preference may be connected to the paternal ability and/or territory quality through competition (Kraak et al., 1999). Furthermore, larger common males in the Netherlands' population invest more sperm into a spawning event compared to smaller males (Zbinden et al., 2001), which may also lead to preference for larger males through fertilization assurance. In our study, we found that white female sticklebacks showed no preference for either male ecotype, while male body size can play a role in female response (albeit weakly in our study) even though overall common females were less likely to respond compared to white females during courtship. When presented with only one stimulus (one male stickleback) female response may represent her absolute preference for a mating partner (Dougherty & Shuker, 2015; Wagner, 1998), if a female rejects a male this may indicate a stronger preference for a different male type since there is the potential that the female would not encounter another male (Dougherty & Shuker, 2015 and references therein). Moreover, this lack of clear preference by females for either male ecotype may arise if both female ecotypes are attracted to the paternal ability provided by common males (van Iersel, 1953), while also being interested in the more energic courtship attempts and bright colouration of white male sticklebacks (Haley et al., 2019; Jamieson et al., 1992a). For white and common female Threespine Stickleback, there are different expectations toward care for their offspring. Because common male sticklebacks provide paternal care and white male sticklebacks do not, there is no expectation of care being provided toward offspring by white females where as there are clear benefits associated with selecting a mate who will provide adequate paternal care for common females. This lack of response by common females and lack of preference by white females is further supported by the result that four of the five hybrid matings were between common males and white females.

For common females to select a mate, they need to be able to assess male quality. In Threespine Sticklebacks, male competition for territories is intense, usually with larger males acquiring the higher quality and larger territory over smaller males (Candolin & Voigt, 2001). Large and high‐quality territory acquisition can be used as a cue to females to show competitiveness (Rowland, 1989c; van den Assem, 1967) and defense against or avoidance of predators (Foster & Ploch, 1990) that are beneficial traits to have when guarding a territory and providing parental care to better support and protect offspring.

Male white stickleback do not perform parental care (Blouw & Hagen, 1990) and thus size may not be an important factor during mating choice. Instead, their energetic courtship attempts and bright colouration (Haley et al., 2019; Jamieson et al., 1992a) might be attractive to females. Common female stickleback may be more attracted to white males as common male stickleback cannot invest as much energy in courtship and nuptial colouration, as it can reduce energy reserves and decrease in egg survival as seen in a population of stickleback from California (von Hippel, 2000). However, other studies examining courtship and hatching success found that in common and 15‐spine sticklebacks males who courted more had higher offspring hatch and/or survival rates (Chiara et al., 2022; Östlund & Ahnesjö, 1998) and were preferred by females (Östlund & Ahnesjö, 1998), but did not find an association between female preference or male parental ability and the size of male 15‐spine sticklebacks (Östlund & Ahnesjö, 1998).

An additional hypothesis that suggests female preference for males who court frequently may be a connection between his sperm availability and courtship vigor (Weir & Grant, 2010). Females may avoid mating with potential sperm‐depleted males in favor of an individual who could fertilize all of their eggs (Harris & Moore, 2005; Nakatsuru & Kramer, 1982). Spermatogenesis in common Threespine Sticklebacks is physiologically inhibited by androgens until breeding ceases at the end of the season (Borg, 1981; Borg & Mayer, 1995). As a result, male stickleback who mate multiple times have significantly reduced sperm counts and smaller ejaculate size compared with virgin males (Zbinden et al., 2001). Thus, females may use their courtship rates as an indicator of quality, which may explain why common females responded to white males. While nothing is known about sperm regeneration in white males, Samuk et al. (2014) hypothesized that white male stickleback may be investing more in gonadal tissues than common male stickleback, as opposed to brain tissue for the purpose of parental care. Further investigation into sperm production of the white stickleback and the connection between sperm availability and courtship vigor are required.

### 4.3 Interbreeding in white and common Threespine Sticklebacks

Ten spawning events occurred during the experiment, resulting in ~29% spawning success. Of these 10 spawnings, five were pure crosses while the other five were hybrid crosses that occurred in both directions and from combinations of both allopatric and sympatric populations. The observation that white and common Threespine Sticklebacks will interbreed is not congruent with the findings from previous studies on sympatric populations of these ecotypes, where mating was completely assortative both in the laboratory and the field (Blouw & Hagen, 1990; Jamieson et al., 1992a).

Reinforcement is related to the prezygotic barriers that aid in selecting against hybrids in sympatric populations (Dobzhansky, 1940; Servedio & Noor, 2003). It is thought that sympatric populations should evolve stronger mating preferences compared with allopatric populations if hybrids are less viable or have reduced fertility, as selection can act on populations in sympatry to favor conspecifc matings (Dobzhansky, 1940; Rundle & Schluter, 1998; Servedio & Noor, 2003 and references therein). While there is evidence that some sympatric populations of Threespine Stickleback ecotype pairs found in Washington, USA have evolved strong mate choice based on male colouration to avoid inbreeding (McPhail, 1969; Scott, 2004), there are instances where populations from the same watersource in Washington show little preference for colouration (McKinnon, 1995; Tinghitella et al., 2015). Similarly, there are some sympatric populations of stickleback that show preferences for specific traits based on ecotype that may also interbreed under certain conditions (Conte & Schluter, 2013; Hagen, 1967; Ólafsdóttir et al., 2006). For example, benthic and limnetic Threespine Sticklebacks are known to hybridize in the wild and do so based on size matching. Benthic stickleback are larger in size and heavier in mass than limnetic stickleback (Nagel & Schluter, 1998; Schluter & McPhail, 1992) and hybridization may occur when individuals on the extreme end of their ecotype's size distribution overlap with the extreme of the other ecotype (e.g., a small benthic with a large limnetic) (Conte & Schluter, 2013; Nagel & Schluter, 1998). However, our results suggest that assortative mating based on size does not occur, as males courted females that were both larger and smaller than themselves. In the Little Campbell River in British Columbia, Canada, pure marine and freshwater stickleback occupy two separate ends of the coastal stream, but there is evidence of hybridization where the two populations connect in the middle (Hagen, 1967). There are also allopatric populations in Washington of red and black ecotypes of Threespine Stickleback that usually mate assortatively but may exhibit inter‐ecotype matings if they cannot choose among potential mates (McPhail, 1969). A similar situation may arise for female stickleback because as time passes, the lifespan of Threespine Stickleback eggs shortens. As a result, to ensure the fertilization of their eggs, female sticklebacks may become less discriminatory of their choice of mates from among their own ecotype (Bakker & Milinski, 1991; Wirtz, 1999). Furthermore, gene flow can occur between the white and common ecotypes (Samuk et al., 2014) despite the observation of an estimated divergence time of ~1 million years (Samuk, 2016). Similar observations have been made for recently diverged (ca. ~10,000 years) Icelandic sympatric stickleback populations for which instances of interecotype spawning events have been observed, despite the general observation that they mate assortatively (Ólafsdóttir et al., 2006).

Because the two ecotypes are interfertile, produce viable offspring, and their identifying traits are heritable (Blouw, 1996), it is logical to expect that there may be instances where hybridization may occur. However, there is evidence in favor of the maintenance of the two ecotypes, where initial findings reported that the offspring of F1 hybrids are not adequate fathers, which results in egg death without artificial aeration (C. Behrens, personal communication). Additionally, one study has shown that the well‐known naturally occurring hybrids of the limnetic and benthic stickleback pair showed a decline in hybridity across three life‐history stages (juvenile, subadult, and adult) and four life cycles (Gow et al., 2007). A generational study would be beneficial to fully understand the impacts of mate selection (or lack thereof) within the common and white ecotypes of the Threespine Stickleback to understand the conditions under which these ecotypes may remain separated over time.

## 5 CONCLUSION

While previous studies have examined female mate choice in populations of both common (Blouw & Hagen, 1990; Milinski & Bakker, 1990; Ridley & Rechten, 1981; Rowland, 1989a) and white (Blouw & Hagen, 1990) Threespine Sticklebacks, less is known about male mate choice in the common stickleback (but see Hagen, 1967; Kraak & Bakker, 1998; Rowland, 1982, 1989a). To our knowledge, male mate choice has not been examined in white stickleback. Our study focused on male mate choice and found that both common and white male ecotypes exhibit a preference for relatively larger females. Common male stickleback courted larger‐bodied common and white females within their respective ecotype size range, while white males courted the largest female overall (common females) more frequently. Furthermore, we found that white females showed no preference for either male ecotype in terms of their probability to respond with characteristic mating behavior, and common females exhibited a slight preference for smaller white males. Additionally, our finding that interecotype matings occurred as frequently as pure‐cross matings differ from previous findings in this system (Blouw & Hagen, 1990). These results merit further research to examine the relationship between preference and size, for both males and females. Hybridization in Threespine Stickleback ecotype complex is not uncommon as similar outcomes have been found in limnetic and benthic Threespine Sticklebacks that are known to hybridize based on preference for similarly sized individuals, but these matings are still less prominent than pure spawning events (Conte & Schluter, 2013; Nagel & Schluter, 1998).

Future studies should consider an experimental design that represent conditions that more closely mimic a natural mating environment, with multiple male and female encounters to fully understand the mating preferences of the two stickleback ecotypes. For this study, males were limited to two females and females were subject to a no‐choice trial design, which hinders mate choice. Moreover, this study focused on visual and behavioral mating signals of common and white Threespine Stickleback. While many studies have focused on these characteristics, comparatively fewer have investigated other stimulants, such as olfactory cues and its associated traits (for example, the role of major histocompatibility complex (MHC genes) on mate selection in Threespine Stickleback). Previous reports suggest that common Threespine Stickleback males from various populations who possess an optimal number of MHC genes not only gain benefits in terms of health but are more attractive to selecting females (Eizaguirre et al., 2009; Jager et al., 2007; Stutz & Bolnick, 2017). However, the influence of MHC genes specifically within the white Threespine Stickleback ecotype and between the common and white ecotypes has yet to be investigated and could potentially be a mechanism that maintains the two ecotypes where they occur.

## AUTHOR CONTRIBUTIONS

**Rachel H. Corney:** Conceptualization (equal); data curation (lead); formal analysis (lead); funding acquisition (supporting); investigation (equal); methodology (equal); project administration (supporting); validation (equal); visualization (lead); writing – original draft (lead); writing – review and editing (lead). **Laura K. Weir:** Conceptualization (equal); formal analysis (supporting); funding acquisition (lead); investigation (equal); methodology (equal); project administration (lead); resources (lead); supervision (lead); validation (equal); visualization (supporting); writing – original draft (supporting); writing – review and editing (supporting).

## CONFLICT OF INTEREST STATEMENT

Both authors do not have a conflict of interest.

## Data Availability

The data that support the findings of this study are openly available in Dryad at https://doi.org/10.5061/dryad.p2ngf1vt7.

## Appendix

**TABLE A1:** Generalized linear model selection for the effects of scaled female size, male ecotype (common or white), and female ecotype (common or white) on the proportion of time spent in proximity to a female during the first phase of the experiment and the adjusted total courtship behavior frequency conducted toward the released female by male Threespine Sticklebacks.

Response variable	Predictor	*k*	AICc	∆AICc	*ω*_*i*_	Pseudo *R* ^2^
Proportion of time	**Female ecotype + female size**	**6**	**−141.4**	**0.00**	**0.203**	**.20**
	**Female ecotype × male ecotype × female size**	**11**	**−140.8**	**0.60**	**0.150**	**.32**
	**Female ecotype + male ecotype + female size**	**7**	**−140.8**	**0.63**	**0.148**	**.22**
	**Female ecotype × female size**	**7**	**−140.5**	**0.90**	**0.130**	**.23**
	**Female ecotype × female size + male ecotype**	**8**	**−140.5**	**0.99**	**0.124**	**.25**
	Male ecotype × female size + female ecotype	8	−138.6	2.88	0.048	
	Female ecotype × male ecotype + female size	8	−138.4	3.04	0.044	
	Female ecotype × female size + male ecotype × female size	9	−138.2	3.26	0.040	
	Female ecotype × male ecotype + female ecotype × female size	9	−138.0	3.40	0.037	
	Female size	5	−136.8	4.66	0.020	
	Female ecotype × male ecotype + male ecotype × Female size	9	−136.0	5.42	0.013	
	Male ecotype + female size	6	−135.9	5.55	0.013	
	Female ecotype × male ecotype + female ecotype × female size + male ecotype × female size	10	−135.5	5.95	0.010	
	(Intercept)	4	−134.7	6.75	0007	
	Male ecotype	5	−133.7	7.72	0.004	
	Male ecotype × female size	7	−133.5	7.99	0.004	
	Female ecotype	5	−133.1	8.32	0.003	
	Female ecotype + male ecotype	6	−132.1	9.35	0.002	
	Female ecotype × male ecotype	7	−129.6	11.79	0.001	
Total courtship behavior	**Female ecotype × male ecotype + male ecotype × female size**	**8**	**874.2**	**0.00**	**0.619**	**.12**
	**Female ecotype × male ecotype + female ecotype × female size + male ecotype × female size**	**9**	**875.7**	**1.43**	**0.302**	**.12**
	Female ecotype × male ecotype × Female size	10	878.3	4.11	0.079	
	(Intercept)	3	921.7	47.52	0.000	
	Female ecotype × female size	6	922.8	48.61	0.000	
	Female ecotype	4	923.9	49.65	0.000	
	Female size	4	923.9	49.70	0.000	
	Male ecotype	4	923.9	49.71	0.000	
	Female ecotype + female size	5	924.0	49.80	0.000	
	Female ecotype × female size + male ecotype	7	925.3	51.06	0.000	
	Female ecotype × female size + male ecotype × female size	8	926.1	51.88	0.000	
	Female ecotype + male ecotype	5	926.1	51.91	0.000	
	Male ecotype + female size	5	926.2	51.96	0.000	
	Female ecotype + male ecotype + female size	6	926.3	52.08	0.000	
	Male ecotype × female size + female ecotype	7	927.3	53.11	0.000	
	Female ecotype × male ecotype + female ecotype × female size	8	927.4	53.18	0.000	
	Male ecotype × female size	6	927.5	53.28	0.000	
	Female ecotype × male ecotype	6	927.5	53.30	0.000	
	Female ecotype × male ecotype + female size	7	928.1	53.88	0.000	

*Note*: Shown above are the number of predictors (*k*), Akaike Information Criterion corrected for small sample sizes (AICc), the difference between the model with the lowest AICc value compared to all other predictors (∆AICc), *ω*_*i*_, the weight of a model relative to the complete model set (*n* = 19), and the marginal pseudo‐*R* ^2^ value for top models. The bolded models are those with the lowest AICc values by a difference of two or more. Models with interactions also contain lower order main effects for the predictors.

**TABLE A2:** Generalized linear mixed model selection for the effects of scaled male size, male ecotype (common or white), and female ecotype (common or white) on the probability of female response to male Threespine Stickleback.

Ecotype combination	Predictor	*k*	AICc	∆AICc	*ω*_*i*_	Pseudo *R* ^2^
Both female and male ecotypes	**Female ecotype + male ecotype**	**5**	**84.9**	**0.00**	**0.199**	**.18**
	**Female ecotype + male size**	**5**	**85.0**	**0.07**	**0.192**	**.17**
	**Female ecotype**	**4**	**85.3**	**0.45**	**0.159**	**.13**
	**Female ecotype × male size**	**6**	**86.1**	**1.26**	**0.106**	**.23**
	**Female ecotype + male ecotype + male size**	**6**	**86.6**	**1.76**	**0.082**	**.19**
	**Female ecotype × male ecotype**	**6**	**86.9**	**1.98**	**0.074**	**.21**
	Female ecotype + male ecotype + male size + female ecotype × male size	7	87.9	2.97	0.045	
	Female ecotype + male ecotype + male size + female ecotype × male ecotype	7	88.7	3.8	0.030	
	Female ecotype + male ecotype + male size + male ecotype × male size	7	89.1	4.23	0.024	
	Male ecotype	4	90	5.1	0.016	
	Male size	4	90.2	5.32	0.014	
	Female ecotype + male ecotype + male size + female ecotype × male size + male ecotype × male size	8	90.4	5.5	0.013	
	Female ecotype + male ecotype + male size + female ecotype × male ecotype + female ecotype × male size	8	90.4	5.53	0.013	
	(Intercept)	3	90.4	5.55	0.012	
	Female ecotype + male ecotype + male size + female ecotype × male ecotype + male ecotype × male size	8	91.2	6.36	0.008	
	Male ecotype + male size	5	91.8	6.88	0.006	
	Female ecotype + male ecotype + male size + female ecotype × male ecotype + female ecotype × male size + male ecotype × male size	9	93	8.14	0.003	
	Male ecotype + male size + male ecotype × male size	6	94.1	9.26	0.002	
	Female ecotype + male ecotype + male size + female ecotype × male ecotype + female ecotype × male size + male ecotype × male size + female ecotype × male ecotype × male size	10	95.2	10.3	0.001	
	Female ecotype + male ecotype + male size + female ecotype × male size + male ecotype × male size	8	90.4	5.5	0.013	
	Female ecotype + male ecotype + male size + female ecotype × male ecotype + female ecotype × male size	8	90.4	5.53	0.013	
	(Intercept)	3	90.4	5.55	0.012	
Common female and common male	**(Intercept)**	**2**	**8.0**	**0.00**	**0.811**	**—**
	Common male size	3	10.9	2.91	0.189	
Common female and white male	**(Intercept)**	**2**	**23.4**	**0.00**	**0.668**	
	**White male size**	**3**	**24.8**	**1.40**	**0.332**	**.11**
White female and common male	**(Intercept)**	**2**	**20.0**	**0.00**	**0.811**	**—**
	Common male size	3	22.9	2.91	0.189	
White female and white male	**(Intercept)**	**2**	**28.4**	**0.00**	**0.813**	**—**
	White male size	3	31.3	2.94	0.187	

*Note*: The first output is the full model, while all others are specific subsets. Shown above are the number of parameters (*k*) for the predictors, Akaike Information Criterion corrected for small sample sizes (AICc), the difference between the model with the lowest AICc value compared to all other predictors (∆AICc), *ω*_*i*_, the weight of a model relative to the complete model set (*n* = 19 and 2, respectively), and the marginal pseudo‐*R* ^2^ value for top models. The bolded models are those with the lowest AICc values by a difference of two or more. Models with interactions also contain lower order main effects for the predictors.

**TABLE A3:** Body size data for the trios for each of the 35 trials observed.

Trial number	Male ecotype	Male standard length	Common female standard length	White female standard length
3	Common	50.12	50.25	40.13
4	White	44.42	48.45	37.39
5	White	37.26	57.19	40.28
6	Common	48.64	51.65	42.52
7	White	45.86	51.03	40.72
8	Common	52.16	50.94	44.78
9	Common	46.58	50.91	41.06
10	White	38.33	53.44	38.47
11	White	46.31	49.49	41.72
12	Common	45.26	54.91	40.63
13	Common	47.92	49.49	40.41
14	Common	47.62	49.55	42.75
15	Common	45.12	51.65	43.48
16	Common	51.23	48.45	40.13
17	Common	47.45	52.07	39.57
18	White	35.45	54.65	39.68
19	White	45.33	54.91	42.62
20	Common	47.62	50.43	42.19
21	Common	49.17	49.26	43.37
22	Common	46.55	53.44	40.72
23	White	43.63	52.07	39.57
24	White	42.97	51.52	39.3
25	White	40.29	49.26	44.78
26	White	41.95	49.55	45.28
27	Common	44.07	44.79	42.32
28	Common	50.97	48.45	40.41
29	White	44.28	54.65	42.47
30	White	46.88	50.94	43.48
31	White	39.96	51.65	41.72
32	Common	48.01	50.94	41.06
33	White	49.87	49.36	42.52
34	Common	39.97	53.44	43.48
35	White	40.01	49.55	40.13
36	Common	51.1	51.65	37.39
37	White	41.32	54.91	40.13

*Note*: Each trio consisted of one common or white male Threespine Stickleback and one common females and one white female. Fish body size was measured as standard length to the nearest 0.01 mm.
